# PTSD-related paradoxical insomnia: an actigraphic study among veterans with chronic PTSD

**DOI:** 10.5249/jivr.v7i2.607

**Published:** 2015-07

**Authors:** Mohammad Rasoul Ghadami, Behnam Khaledi-Paveh, Marzieh Nasouri, Habibolah Khazaie

**Affiliations:** ^a^Sleep Disorders Research Center, Kermanshah University of Medical Sciences, Kermanshah, Iran.

**Keywords:** Sleep disturbance, PTSD, Actigraphy

## Abstract

**Background:**

Sleep disturbance is a common self-reported complaint by PTSD patients. However, there are controversies in documenting objective indices of disrupted sleep in these patients. The aim of the present study was to assess sleep disturbances in veterans with chronic PTSD, using both subjective and objective assessments.

**Methods:**

Thirty two PTSD patients with complaints of insomnia were evaluated using the Clinician Administrated PTSD Scale version 1 (CAPS) and completed the Pittsburg Sleep Quality Index (PSQI) for subjective evaluation of their sleep. For objective evaluation, participants underwent two consecutive overnight actigraphic assessments. Total Sleep Time (TST), Sleep Latency (SL), Sleep Efficiency (SE) and Number of Awakening (NWAK) were measured in all participants.

**Results:**

Participants underestimated TST (p less than 0.0001), SE (p less than 0.0001) as well as NASO (0.03) in the questionnaire compared to the actigraphic assessment and overestimated SL (p less than 0.0001).

**Conclusions:**

Objective sleep parameters do not adversely affect veterans with chronic PTSD. Self-reported sleep disturbance in these patients is not reliable and objective sleep assessments are necessary.

## Introduction

Insomnia and sleep disturbance, which cause considerable subjective distress and treatment resistance,^1^ are common complaints among patients with post-traumatic stress disorder (PTSD). There is abundant evidence of the association between PTSD and sleep disturbances, based on self-reported symptoms and questionnaires.^2^ Also, sleep disturbances can be found in both the hyper arousal and re-experiencing clusters of symptoms in the DSM-IV-TR.^3^

The most frequent self-reported complaints by PTSD patients are difficulties initiating and maintaining sleep, shorter sleep duration, restless sleep, and especially nightmares.^2^

Studies demonstrated that 44% of veterans with PTSD reported difficulty falling sleep. Ninety-one percent had difficulty maintaining sleep and 52% reported nightmares.^4^

Several studies showed sleep disturbance in PTSD patients; however, there are controversies about studies using objective sleep measurements to record markers of disrupted sleep in these patients. Some polysomnographic (PSG) studies showed impairments in some sleep parameters in PTSD patients,^5^ whereas other studies using PSG ^6^ and actigraphy ^7^ showed no significant differences in sleep parameters between subjects with and without PTSD.

There are a few studies that examined differences between subjective and objective sleep measurements, such as sleep efficiency and nocturnal awakening, in veterans with PTSD.^6,8^ In our clinical experience, veterans with chronic war-induced PTSD complained fervently of insomnia but they had normal sleep patterns in objective (polysomnography or actigraphy) assessments.

Due to the apparent discrepancy in findings about sleep in individuals with PTSD, the aim of the present study was to evaluate subjective and objective assessments of sleep parameters in veterans of the Iran-Iraq war diagnosed with chronic PTSD.

## Methods

This study was approved by the research council and ethics board of Kermanshah University of Medical Sciences (KUMS) in Kermanshah, Iran, and was conducted from November 2012 to August 2013 in the Sleep Disorders Research Center of KUMS. It compared subjective and objective (actigraphy) sleep recordings in veterans of the Iran-Iraq war (1980 to 1988) diagnosed with chronic PTSD (n=32). A psychiatric interview using the Structured Clinical Interview for the DSM (SCID) was completed for all participants. PTSD was defined by criteria described within the DSM-IV-TR. All participants reported trauma related to combat experience, bombardment, or mine explosion.

All participants presented with complaints of insomnia. After psychiatric interview, patients with other psychiatric disorders, psychotic disorders, malingering and other underlying diseases were excluded from the study. Also, participants with sleep apnea or periodic limb movement during sleep were excluded. All participants were evaluated by a well-trained clinical psychologist using the Clinician Administered PTSD Scale version 1 (CAPS).

The CAPS is a structured clinical interview aimed at uncovering core and associated symptoms of PTSD. The combat traumatic event (the index event) reported by the participant was evaluated at the beginning of the interview to determine whether it met the diagnostic criteria for criterion A (traumatic event). If the index event met the criteria, frequency and intensity of 17 PTSD symptoms were rated on 5-point scales ranging from 0 (Never [frequency], Not at all [intensity]) to 4 (daily or almost daily, extremely). When the participant reported at least the frequency rating of 1 and the intensity rating of 2 for the symptom; the symptom was considered as positive.^9,10^

For subjective evaluation of sleep, participants completed the Pittsburg Sleep Quality Index (PSQI). The PSQI is a validated questionnaire that estimates overall sleep quality. This scale has seven domains (sleep quality, sleep latency, sleep duration, sleep efficiency, sleep disturbances, use of sleep medications and daytime dysfunction). A Persian version of PSQI was validated in PTSD patients with diagnostic sensitivity of 100% and specificity of 93%. Overall the Cronbach’s alpha coefficient was 0.89.^11^

For objective evaluation, participants underwent two consecutive overnight actigraphic assessments. The actigraph is a portable device (similar to a wrist watch) that records patient movement in order to evaluate sleep parameters such as total sleep time, sleep onset latency, total wake time, number of awakenings, and sleep efficiency.^12-15^ Participants wore an ambulatory Monitoring Actigraphy (Somnomedics, Germany) on the wrist of their non-dominant hand. Participants followed their habitual sleeping habits as closely as possible. Data recorded by actigraphy were then downloaded to a computer and the average of variables over two nights was used for analysis.

Statistical analysis: Data from the assessment instruments were tabulated and the normal distribution of data was verified using the Kolmogrov-Smirnov test. Paired t-test and chi square test were used to compare results using SPSS v. 19.0. Correlation between self-reported sleep parameters and those estimated by actigraphy was obtained using Pearson correlation coefficient. P values less than 0.05 were considered statistically significant.

## Results

Thirty-two volunteers (mean age 45.7 ± 14.9) met the criteria for participation in this study. Demographic characteristics are shown in Table 1.

**Table 1:** Participant characteristics.

	Participants (n=32)
**Age (year)**	50.4±6.5
**BMI (kg/m2)**	25.7±5.7
**Current smoker**	15 (46)
**Substance abuser**	6 (18)
**Drugs**	
**Anti-hypertension**	14 (43)
**SSRI**	16 (50)
**TCA**	9 (28)
**Benzodiazepine**	18 (56)
**Anipsychotics**	12 (37)

Data presented as mean ± SD or number (%)

Table 2 presents means and standard deviations of Total Sleep Time (TST), Sleep Latency (SL), Sleep Efficiency (SE) and Number of Awakening (NWAK) measured by PSQI and actigraphy for the 32 participants.

**Table 2:** Summary of questionnaire- and qctigraphy-measured sleep parameters.

	PSQI questionnaire	Actigraphy	p value
**TST (hour)**	4.2±1.9	7.1±1.2	<0.0001
**SL (min)**	75±51	20.3±19.8	<0.0001
**SE (%)**	59.3±24.8	81.2±10.6	<0.0001
**NWAK (n)**	4.1±2	10±7.4	0.003

Data presented as mean ± SD

Average total sleep time reported by participants in the questionnaire ranged from 0 to 10.0 h (mean 4.2±1.9 h). Actigraphy measures demonstrated that total sleep time ranged from 4.31 hrs to 8.6 hrs (mean 7.1±1.2 h). Comparison of average measures for TST ratings across the two assessment tools showed significant differences between self-reported and actigraphy measurements (p<0.0001). 

Similar results were observed for sleep efficiency. Based on the PSQI finding, the mean sleep efficiency of participants was 59.3±24.8%. However, actigraphy assessment demonstrated 81.2±10.6% for sleep efficiency, revealing greater sleep efficiency than was subjectively estimated in the PSQI (p <0.0001).

We also found that participants overestimated sleep latency compared to the actigraphy results. Mean time to falling asleep reported in the PSQI was 75.7±51 min, which was significantly more than the time estimated by actigraphy (20.3±19.8 min) (p<0.0001).

Also, the sleep questionnaire revealed that participants awoke between 0 and 10 times per sleep period. Mean self-reported wake episodes were 4.1±2 per period of sleep. However, actigraphy recordings showed that awakenings were more frequent and ranged from 2 to 48 per sleep period (mean 10±7.4) (p=0.003).

In addition, there was no significant correlation between self-reported sleep parameters and those estimated by actigraphy (p>0.05).

## Discussion

In this study, we compared subjective and objective sleep characteristics using PSQI and actigraphy in veterans of the Iran-Iraq war diagnosed with chronic PTSD. We found subsequent differences in sleep parameters between PSQI assessments and actigraphy recordings that revealed paradoxical insomnia in our PTSD subjects.

Our findings showed that mean total sleep time and sleep efficiency reported by participants were lower than that recorded by the actigraphy. Moreover, actigraphic measurements showed that participants had less sleep latency than they have claimed in the questionnaire.

Although several studies have been conducted on the relationship between subjective and objective sleep parameters of PTSD patients,^6-9^ our findings showed gross discrepancies. Our results showed that despite subjective complaints of insomnia and sleep problems by veterans with chronic PTSD, subjects had normal actigraphic records. This is the hallmark of diagnosis for paradoxical insomnia according to the International Classification of Sleep Disorders (ICSD).^16^

Paradoxical insomnia is common among PTSD patients. Leskin et al, for example, reported that 80% of PTSD patients suffered from paradoxical insomnia.^17^

Studies showed that sleep logs in PTSD patients have low reliability comparing to actigraphy recordings. Westermeyer et al showed that PTSD patients reported significantly less total sleep time than were recorded by actigraphy. Further, they observed that in PTSD patients there were significantly fewer self-reported wake episodes than actigraphic wake episodes (2.7 ± 2.5 self-reported awakenings per sleep period vs. 4.1 ± 2.7 wake episodes per sleep period by actigraphy).^8^ This is similar to our finding that revealed 4.1±2 self-reported awakenings per sleep period vs. 10±7.4 wake episodes in actigraphy records.

This finding would seem in conflict with Germain and Nielsen’s study that reported more nocturnal awakenings among PTSD patients. However, PTSD participants in their study had elevated periodic leg movements (PLM) indices and the authors suggested that PLM may be correlated with processes contributing to intense negative dreaming, hyperarousality and nocturnal awakenings among these patients.^18^ Also, Lavie et al., in their general observation, reported that sleep complaints exceeded objective findings in PTSD-related insomnia.^19^

Higher nocturnal awakening in actigraphy recordings compared to self-reported recordings may be also due to the over-reporting of awakenings by actigraphy, because individuals with PTSD move sufficiently while asleep to record an erroneous awakening response in the actigraphy.^8^

Our explanation for these findings could be that the dramatic perception of insomnia in war-induced chronic PTSD may be due to the amplification of brief arousals from sleep (REM sleep). Another explanation for these findings may be impaired memory of the awakening episode by PTSD sufferers.^8^

In spite of the fact that paradoxical insomnia is a subtype of chronic insomnia, few studies have been done on its etiology and no standard treatment has been suggested for this condition.^20^

It seems that, in addition to the physiological mechanisms, like high levels of sympathetic activation of the autonomic nervous system^21^ or dysfunction of the hypothalamic-pituitary-adrenal axis, which are associated with the symptoms, some other cognitive and behavioral mechanisms may play a role in pathophysiology of paradoxical insomnia.^22^

The effect of anxiety, ruminative thoughts and some personality traits has been considered in the provoking and continuation of this condition.^23^

The difference between objective and subjective sleep assessments in PTSD patients may be due to the method of PSG, since insomnia occurs with high levels of anxiety and PSG decreases the level of anxiety (reassuring effect for being monitoring by PSG and actigraphy and inducing a cognitive and emotional state) and can decrease the insomnia.^24^

Moreover, one explanation for this difference could be that current objective measurements of sleep analysis may not diagnose all the sleep problems of PTSD patients; therefore, it is not reasonable to claim that all intricate aspects of sleep mechanism can be analyzed by PSG and actigraphy.

Although our sample was small and selected, our findings revealed an overestimation of sleep disturbances by veterans with chronic PTSD, which is consistent with prior reports that PTSD patients overestimated their sleep disturbances. Based on these findings, we suggest that self-reported sleep disturbance in veterans with chronic PTSD is not reliable and objective sleep assessments for these patients are necessary. It must be noted that severe complaints of insomnia may result in the consumption of hypnotic drugs (e.g. benzodiazepines) with or without prescription. Hypnotic drugs may have mild to moderate effects on improvement of insomnia state and are only recommended for use in the short-term because of the risk of dependency or abuse. In severe cases where the patients are adamant that they suffer from insomnia despite normal objective findings, we may suspect overvalued ideation or somatic-like delusion and so cognitive-behavior therapy (CBT) and atypical antipsychotic drugs may be considered as appropriate treatments.^25,26^

This study has several limitations. The sample size was small. Moreover, we used only two consecutive overnight actigraphic recordings, which may be insufficient to adequately assess sleep/wake patterns.

## Conclusion

Thus, considering all of these findings together, except for nocturnal awakening, objective sleep architecture does not adversely affect veterans with chronic PTSD. Nevertheless, these patients fervently complain of insomnia and sleep problems. We suggest that because of unreliable self-reported sleep disturbance, objective sleep assessments for veterans with chronic PTSD are necessary.
